# Willow silvopastoral systems as a strategy to reduce methane emissions while maintaining cattle performance

**DOI:** 10.1038/s41598-025-02289-0

**Published:** 2025-06-02

**Authors:** Joshua Philip Thompson, Sokratis Stergiadis, Omar Cristobal Carballo, Wayne E. Zeller, Tianhai Yan, Francis Lively, John Gilliland, Rudra N. Purusottam, Sharon Huws, Katerina Theodoridou

**Affiliations:** 1 Institute of Global Food Security, Queen’s University Belfast, Belfast, UK; 2 School of Biological Sciences, Queen’s University Belfast, Belfast, UK; 3 School of Chemistry and Chemical Engineering, Queen’s University Belfast, Belfast, UK; 4 School of Agriculture, Policy and Development, University of Reading, Reading, Berkshire UK; 5 Sustainable Livestock Systems Branch, Agri Food and Biosciences Institute, County Down, Hillsborough, UK; 6 USDA-ARS, U.S. Dairy Forage Research Center USA, Madison, UK; 7 Brook Hall Estate, Londonderry, Northern Ireland, UK

**Keywords:** Willow, Silvopasture, Condensed tannins, Methane, Performance, Cattle, Climate-change mitigation, Animal physiology

## Abstract

Willow (Salix sp.) is a common tree used in agroforestry for biofuel production and contains condensed tannins (CTs). This study investigated, for the first time, the feasibility of using willow grazing in a silvopastoral system to improve ruminant productivity while minimizing environmental impact. The specific objectives were to: (a) characterise willow’s CTs, and (b) explore their effect on methane emissions and animal performance. Twenty growing castrated beef cattle (steers) were used in a two-treatment study comparing grazing systems: a willow fodder mix with a grass understory (WFG) and perennial ryegrass grazing (PRG). The study was conducted using a two-period Latin square design. Steers grazing on WFG had an average daily CT intake of 617 g/d. For CTs, the mean degree of polymerisation was 10.6, and the ratio of procyanidin to prodelphinidin was 28.9:71.2. No significant differences were observed between the two treatments in metabolisable energy intake (*P* = 0.0728), nitrogen intake (*P* = 0.844), forage dry matter intake (*P* = 0.100), or total dry matter intake (*P* = 0.0591). A 27% reduction in methane production was observed for the WFG treatment relative to PRG (*P* < 0.001; 173 vs. 273 g/d). This study is the first to provide evidence of the significant potential that willow could have in sustainable livestock production systems worldwide.

**Supplementary Information:**

The online version contains supplementary material available at 10.1038/s41598-025-02289-0.

## Introduction

Due to the intensification of ruminant livestock systems, shrubs and trees have been gradually removed from pastoral landscapes and replaced with permanent grasslands^1^. While these changes have improved efficiency and productivity, they have come at the expense of ecosystem biodiversity^2^. Silvopastoralism, one of the most ancient forms of agroforestry, uses trees and shrubs to feed ruminants as part of a multifunctional land-use system integrated alongside pasture^3,4^. Silvopasture provides numerous environmental benefits, including carbon dioxide (CO₂) sequestration, enhanced biodiversity, and improved soil fertility, while also reducing soil erosion. Leaves of trees and shrubs provide energy, protein, and a range of beneficial secondary metabolites, which can offer significant nutritional and medicinal benefits for animals^3^.

Willow (Salix sp.) is a common tree used in agroforestry for biofuel production, intended as a renewable energy source. However, during that process, leaves and branches less than 18 mm in diameter (tree fodder) are considered waste and are often discarded^5^. These by-products, however, could be put to various uses, contributing to a more sustainable and resource-efficient system that aligns with a circular economy framework. Additionally, these waste by-products have potential applications across different industries, such as animal feed, supplements, and functional foods, due to their medicinal properties, such as salicin^5^. By utilising willow by-products, they can be transformed into valuable resources within a regenerative and sustainable agricultural system, promoting sustainable biomass production and supporting carbon sequestration.

Willow tree fodder also has a high protein content (values > 20%) and contains a class of plant secondary compounds: condensed tannins (CTs)^5^. Currently, there is significant interest in the inclusion of CTs in ruminant nutrition due to their potential to mitigate the environmental impacts of livestock^6^. This interest stems from the fact that ruminants are responsible for 14% of anthropogenic methane (CH₄), a potent greenhouse gas (GHG) produced by rumen methanogens^7^. Furthermore, ruminants excrete around 70% of ingested nitrogen (N), which can be converted by denitrifying soil bacteria into nitrous oxide (N₂O), a GHG with a warming potential 298 times greater than CO₂. Alternatively, nitrogen excreted in urine can react with faecal ureases to release ammonia (NH₃), with livestock accounting for 22% of global agricultural NH₃ emissions^8–11^. Ammonia release is significant because it contributes to air and water pollution, soil acidification, and ecosystem eutrophication. Therefore, combining CT-containing willow silvopastoral systems with ruminant livestock has the potential to reduce the environmental impact of ruminant production.

To date, available data on the effects of CTs on methanogenesis suggest their mode of action includes: (a) the inhibition of methanogenic bacteria and archaea in the rumen^12^, and (b) an indirect effect on hydrogen availability (a substrate for CH₄ production) through the binding of CTs to fibre in the rumen, which depresses its degradation^13,14^. However, the structural composition of CTs affects their properties and is believed to influence how they affect digestion and the nutritional value of feed^15^. The structural chemistry of CTs, such as mean degree of polymerisation (mDP), prodelphinidin: procyanidin (PD: PC) ratio, and cis: trans flavan-3-ol ratios, is correlated with a greater ability to complex with proteins or carbohydrates, which affects ruminal CH₄ production^16,17^. Within optimal ranges (< 3% DM), CTs have been shown to improve animal productivity by binding with protein in the rumen (bypassing fermentation) and later dissociating in the abomasum, making more protein available for absorption and growth^18,19^. Consequently, utilising CTs present in willow within a silvopastoral system could help mitigate environmental challenges associated with the ruminant livestock sector.

Therefore, this study aimed to evaluate, for the first time, the potential of grazing willow within a silvopastoral system to enhance sustainable ruminant production. The specific objectives were to: (a) characterise willow’s condensed tannins, and (b) predict CT intake and assess its effect on CH₄ emissions and animal performance.

## Results

### Willow fodder and grass understory forage dry matter yield

Table 1 shows that the DM yield of each weekly block was 51% lower for willow fodder (WF) during period 2 (P2) compared to period 1 (P1) (pairwise t-test, *P* < 0.010). Overall, the DM yield of willow fodder grass (WFG) was 59% lower in P2 than in P1 (pairwise t-test, *P* < 0.05). No differences were observed between periods in terms of the proportion of WF from the total WFG available (pairwise t-test test, *P* > 0.10).

**Table 1:** Estimated forage dry matter yields (kgDM) of the Willow forage grass site and the percentage of Willow fodder in the treatment forage.

	Period		s.e.m.	P-value
	1	2		
	(*n* = 12)	(*n* = 12)		
WF (kgDM)	625	304	67.2	**
GU (kgDM)	269	60	61.8	0.091
WFG (kgDM)	895	364	125.3	*
WF available (%)	73	83	33.4	0.130

*WF* willow fodder,* GU* grass understory,* WFG* willow fodder grass, the symbol: ‘*’,’**’, denote significance P < 0.05 and 0.01 respectively. the ‘n’ value in the table represents the number of biological replicates.

### Condensed tannins content and structure

From the sequential HBAI assays on WF samples from P1 and P2 (Table 2) there is no differences in total CTs content between periods. However, the unbound CTs content increased by 13% in P2 (pairwise t-test; *P* < 0.05). For this study, we are concerned with the unbound CTs as these are soluble and are likely available for interactions with biomolecules in the ruminant’s digestive tract, including protein.

**Table 2:** Condensed tannin content and structure in Willow fodder and faeces.

	Period		s.e.m.	P-value
	1	2		
Willow Fodder CT content (%DM)	(*n* = 9)	(*n* = 9)		
Bound	5.51	5.25	0.116	0.310
Unbound	7.30	8.25	0.233	*
Total	12.8	13.5	0.245	0.170
Willow Fodder CT structure	(*n* = 1)	(*n* = 1)		
Mean degree polymerisation	10.8	10.3		
Procyanidin content (%)	28.7	29.0		
Prodelphinidin content (%)	71.3	71.0		
*Cis* content (%)	22.8	23.9		
*Trans* content (%)	77.2	76.1		
Steer faecal CT content (%DM)	(*n* = 6)	(*n* = 6)		
Bound	1.97	1.52	0.153	0.160
Unbound	blq	blq		
Total				

*CT* condensed tannin,* Blq* below limit of quantification. The symbol: ‘*’,’**’, denote significance P < 0.05 and 0.01 respectively. The ‘n’ value in the table represents the number of biological replicates.

**Fig. 1:**
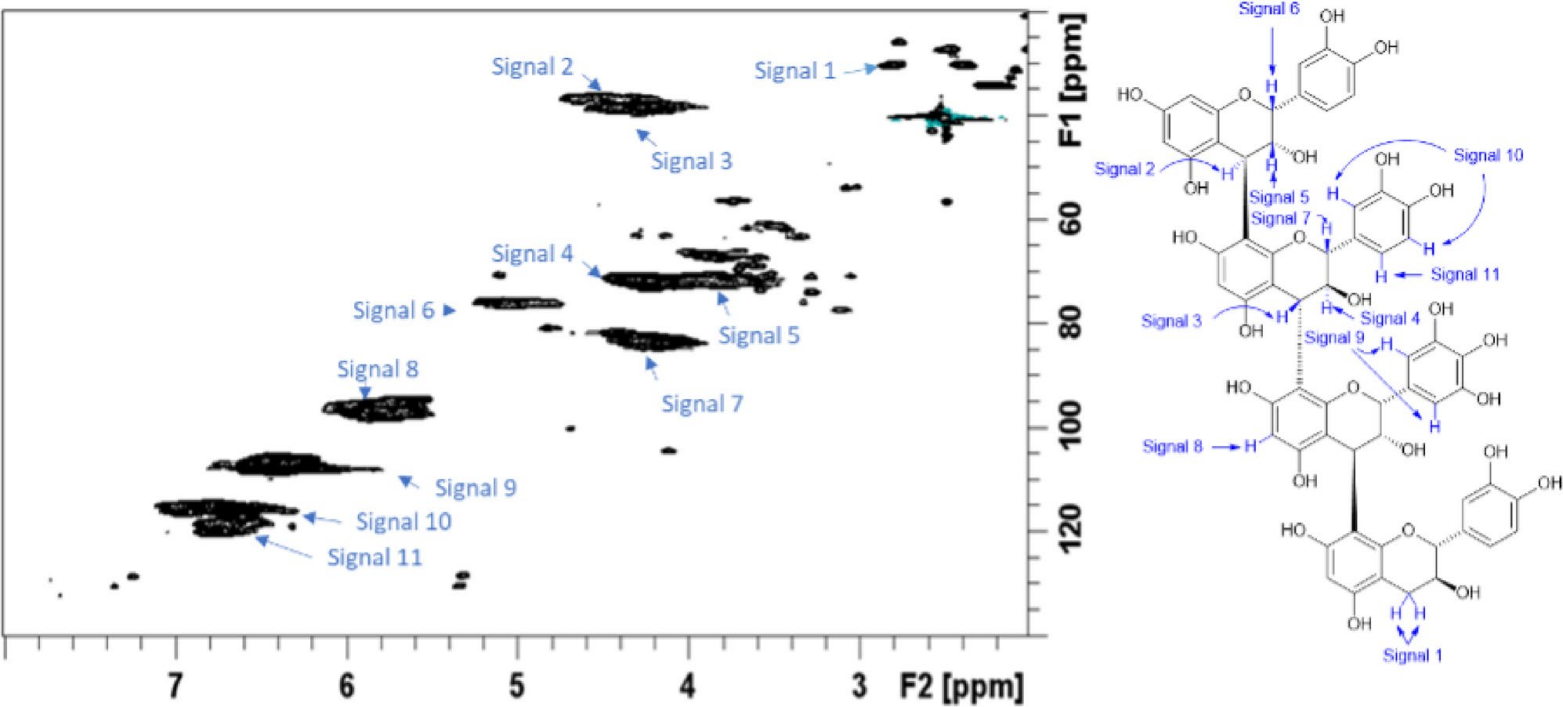
^1^-^13^ C HSQC NMR spectrum of willow fodder CT reference standard from period 1 with signals identified as listed in the accompanying condensed tannin structure. .

**Fig. 2:**
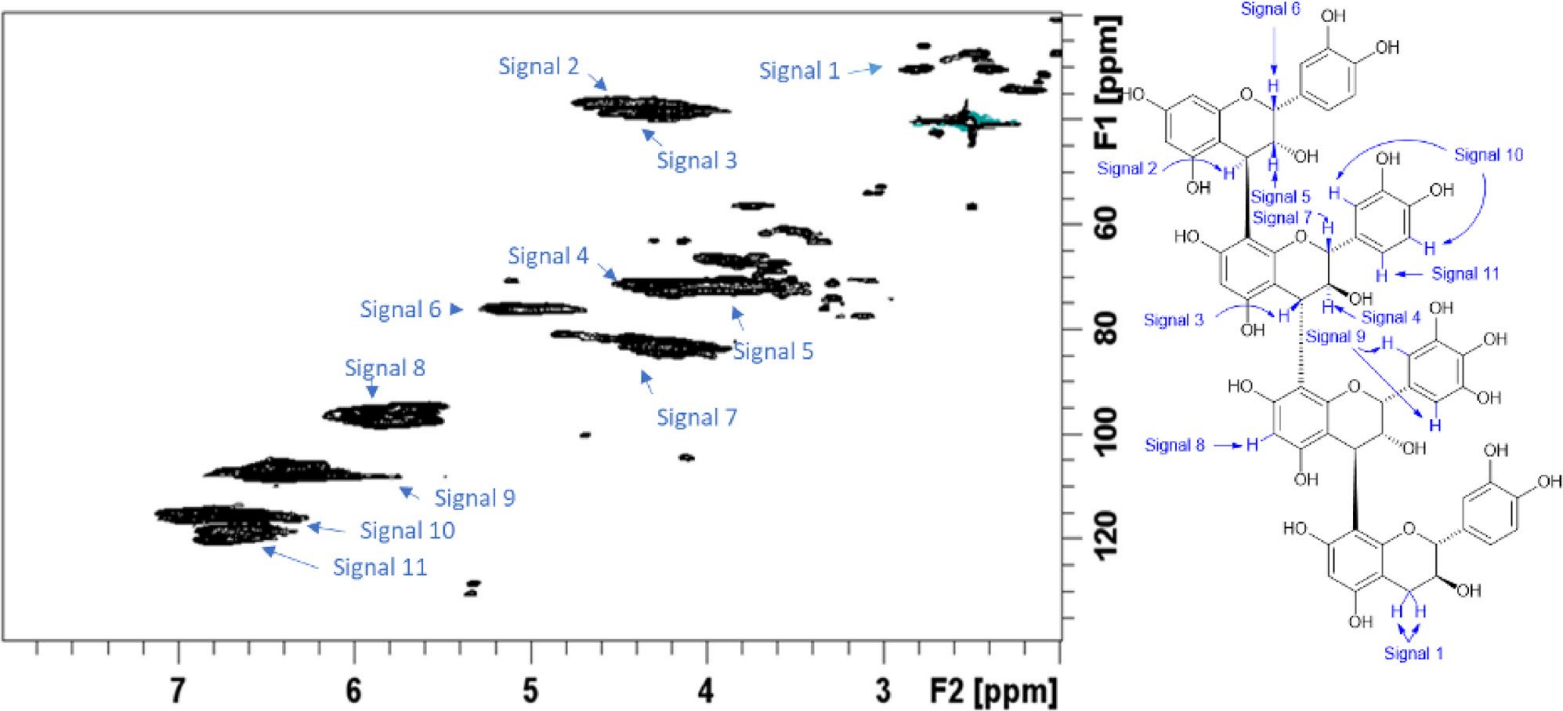
^1^H-^13^C HSQC NMR spectrum of Willow fodder CT reference standard from period 2 with signals identified as listed in the accompanying condensed tannin structure.

Figures 1 and 2 show the ^1^-^13^ C HSQC NMR spectrum of the purified WF CTs reference standard from P1 and P2 respectively. The composition of the CT reference standard for P1 was found to have a PC/PD (procyanidin/prodelphinidin) ratio of 28.7/71.3 (± 0.3), a C-2/C-3 *cis*/*trans* ratio of 22.8/77.2 (± 1.8)^20^ and a mean degree of polymerization (mDP) of 10.8 (± 0.3)^21^. Similarly, for P2 the composition of the CT reference standard was found to have a PC/PD ratio of 29.0/71.0 (± 0.4), a C-2/C-3 *cis*/*trans* ratio of 23.9/76.1 (± 0.4), and a mDP of 10.3 (± 0.1). A small amount of A-type interflavan-3-ol linkages (~ 1%) was detected in both samples. The purity of the purified WF CTs reference standard samples was determined to be approximately 96% and 97% for P1 and P2 respectively (Supplementary Figure S1 and S2). This was based on the relative integration of the impurity cross-peaks arising from carbohydrate anomeric centre and olefinic H/C cross-peak signals in the NMR spectra versus the C-6/8 H/C cross-peak signals from the A ring of the CTs present.

### Feed Intake and dietary nutrient intake

**Fig. 3:**
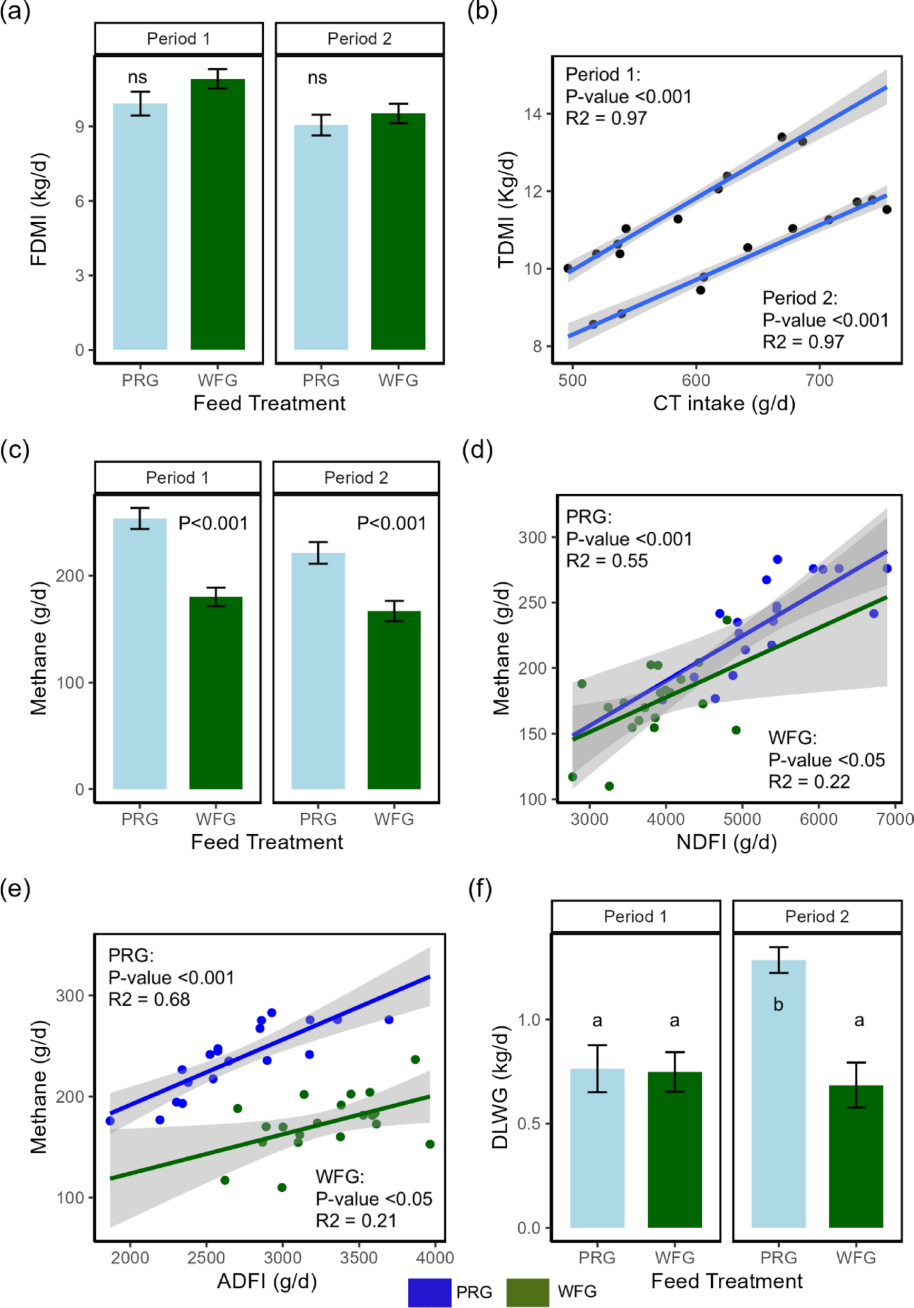

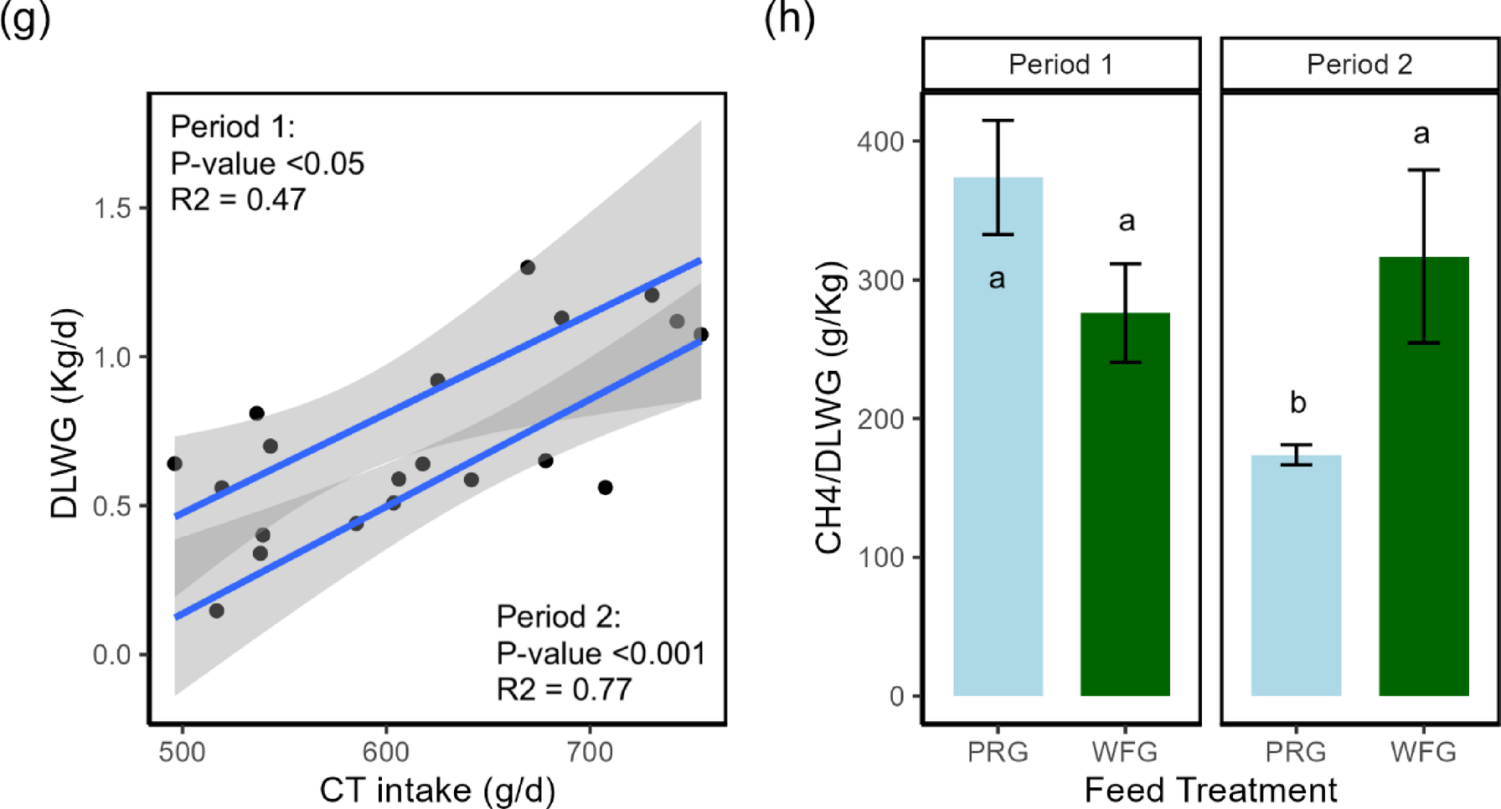
Factors affecting CH_4_ production and live weight gain of steers. (**a**) Stacked bar chart of average forage dry matter intake for each treatment and period. (**b**) Linear model between total dry matter and condensed tannin intake. (**c**) Stacked bar chart of average CH_4_ production for each treatment and period. (**d**) Linear model between neutral detergent fibre intake and methane (g/d). (**e**) Linear model between acid detergent fibre intake and methane (g/d). (**f**) Stacked bar chart of average daily live weight gain of steers for each treatment and period. (**g**) Linear model between average daily live weight gain and condensed tannin intake of willow treatment. (**h**) Stacked bar chart of average CH_4_/DLWG for each treatment and period. Annotation of a and b denotes a significant difference *P* < 0.05.

 All results for feed and dietary nutrient intake are presented in Supplementary Table S2. Total condensed tannin intake (TCTI; g/h/d) and inclusion (CTI; %DM) were greater for the WFG treatment (TCTI = 617 g/d, CTI = 5.65% DM; Kruskal Wallis; *P* < 0.001; Supplementary Table S2, rows 16 and 17). Figure 3a shows that predicted forage DM intake (FDMI: kgDM/h/d) was non-significant between forage treatments; however, an 11% reduction in predicted FDMI occurred in P2 compared to P1 (ANOVA; *P* < 0.010). Overall, there were no differences in total DM intake (TDMI; kgDM/h/d) between forage treatments (ANOVA; *P* = 0.059), periods (ANOVA; *P* = 0.164), or interaction (ANOVA; *P* = 0.274). For the WFG treatment, the relationship between TDMI and TCTI showed a strong positive correlation (linear model; Fig. 3b; *P* < 0.001, R² = 0.97). In terms of energy, GEI was 13% greater for WFG (ANOVA; *P* < 0.01; Supplementary Table S2, row 10), while no differences were observed in total MEI (ANOVA; *P* = 0.073; Supplementary Table S2, row 11). Acid detergent fibre intake (ADFI; kgDM/h/d) was 21% greater (ANOVA; *P* < 0.001; Supplementary Table S2, row 12) for WFG. In contrast, neutral detergent fibre intake (NDFI; kg/h/d) was 40% greater (ANOVA; *P* < 0.001; Supplementary Table S2, row 13) for PRG treatment. No differences in starch or nitrogen intake were observed between treatments (Supplementary Table S2, rows 14 and 15).

### Heat production and methane emissions

Heat production (HP; MJ/d) was non-significant between forage treatments (ANOVA; *P* = 0.384; Supplementary Table S3, row 7). While H₂ production (g/d) was non-significant between treatments, WFG showed a 22% numerical reduction (ANOVA; *P* = 0.088) compared to PRG (Supplementary Table S3, row 4). Alternatively, CH₄ production (g/d) was 27% lower (ANOVA; *P* < 0.001) for the WFG compared to the PRG treatment (Supplementary Table S3, row 5), with Fig. 3c visualising this reduction in both periods. Gross energy lost as CH₄ (CH₄-E; MJ/d) was 27% lower (ANOVA; *P* < 0.001; Supplementary Table S4, row 11) for the WFG compared to the PRG treatment. Likewise, the proportion of energy lost as CH₄-E as a percentage of total GEI (CH₄-E/GEI; %) was 35% lower (ANOVA; *P* < 0.001) for the WFG treatment relative to PRG. Relationships between CH₄ production and NDFI (Fig. 3d) were much stronger with the PRG treatment, while the relationship was significant for the WFG, the R² value was 0.22 compared to 0.55 for PRG. For CH₄ production and ADFI (Fig. 3e), there was a much clearer separation between treatments, with WFG having lower CH₄ than PRG at the same ADFI (Fig. 3e). In terms of CH₄ production and LWG (CH₄/DLWG), forage treatment was non-significant (PRG = 269, WFG = 296; Kruskal Wallis; *P* = 0.555; Supplementary Table S3, row 10). Figure 3h shows the impact of the interaction, which had a significant effect (Kuskal Wallis; *P* < 0.010), with PRG2 (174 g/kg) having a 115% lower (Kruskal Wallis; *P* < 0.050) CH₄/DLWG than PRG1 (374 g/kg), while the rest of the interactions, including WFG1 (276 g/kg) and WFG2 (317 g/kg), were non-significant.

### Animal live weight gain

 The WFG treatment had a 31% lower (ANOVA; *P* < 0.010) DLWG compared to PRG (Supplementary Table S2, row 3). Figure 3f shows that the interaction was significantly different (ANOVA; *P* < 0.010), with the DLWG in the PRG treatment in P2 (PRG2) being 69% greater (pairwise t-test; *P* < 0.050) than PRG1, while the other interactions between PRG1, WFG1, and WFG2 were non-significant (PRG1 = 0.764, WFG1 = 0.748, PRG2 = 1.29, and WFG2 = 0.685). For the WFG treatment, DLWG increased in accordance with CTs intake (Fig. 3g).

### Faecal condensed tannin content and structure

**Fig. 4:**
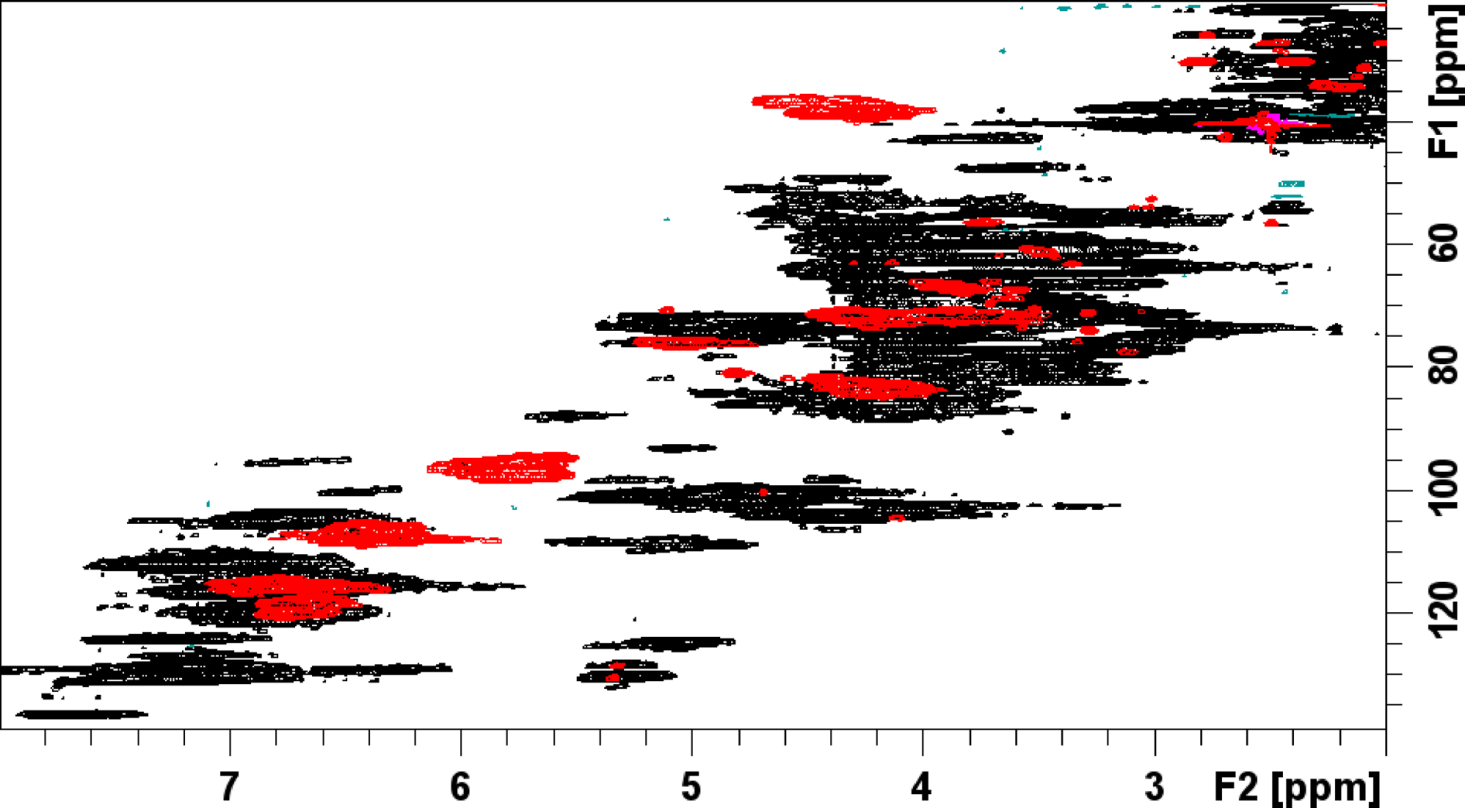
^1^-^13^ C HSQC NMR spectrum of pooled faecal sample of steers grazing WFG in period 1 (black) overlapped with the spectrum of the CT reference standard from Willow fodder period 2 (red).

**Fig. 5:**
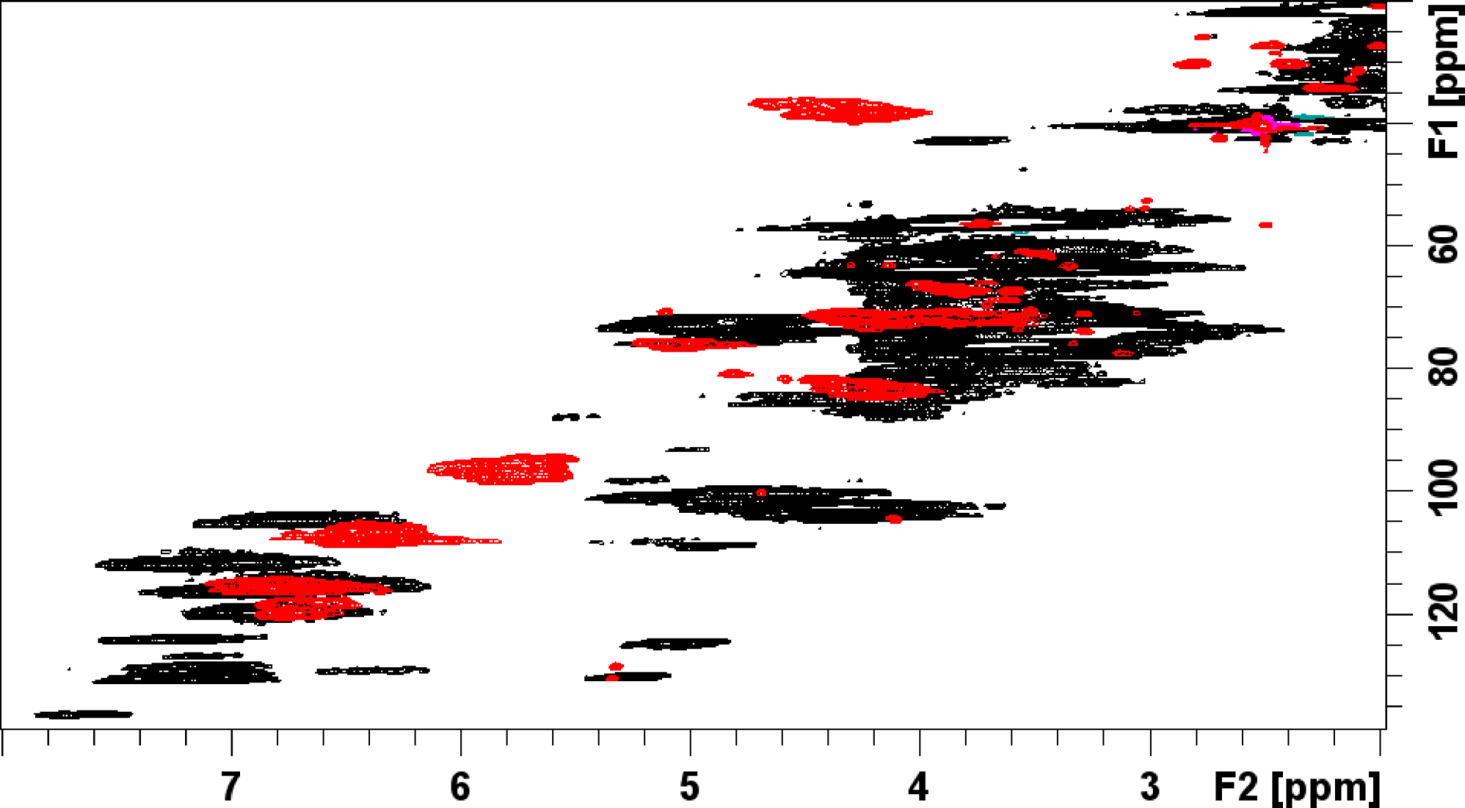
^1^-^13^ C HSQC NMR spectrum of pooled faecal samples of steers grazing WFG in period 2 (black) overlapped with the spectrum of CT reference standard from Willow fodder period 2 (red).

Using the HCl-butanol-iron-acetone assay, no colour was detected in the faecal samples of PRG1 and PRG2 as expected. These faecal samples were derived from PRG with no CTs present. Both the willow fodder samples: WFG1 and WFG2 exhibited a colour change and were generated from the bound fraction of the faeces while no colour was detected in the unbound fractions. Quantification of the colour generated from bound fraction of WFG1 and WFG2 faecal samples versus the CTs reference standard from willow fodder P1 indicated a CT concentration of 1.97 ± 0.22 and 1.52 ± 0.18, respectively. However, examination of these samples via ^1^-^13^ C HSQC NMR spectroscopy shows the absence of intact, unmodified CTs for faecal sample WFG1. Overlap of the 2D NMR spectrum of faecal sample WFG1 (Fig. 4, black) with the 2D NMR spectrum of CTs reference standard from WF period 2 (Fig. 4, red) emphasizes the absence of key CTs cross-peaks signals. Likewise, Fig. 5 shows the same for faecal samples from WFG2. Thus, the colour seen in the bound fraction of the sequential HBAI assay is not derived from intact CTs. The colour may be derived from some CT metabolite(s), or some other components generated during the ruminant digestive process and is unknown.

## Discussion

Despite willow’s well-recognized role in the biorenewable energy sector, its promising contributions towards sustainable ruminant production, whether integrated within silvopastoral systems or utilized as tree fodder, have not been studied in the context of our journey towards net-zero carbon goals. This study is the first to concurrently evaluate its impact on both animal performance and CH₄ emissions within a grazing context.

### Willow silvopastoral systems can be rotationally grazed but require a longer grow back period than perennial ryegrass 

Generally, PRG systems require a grazing interval between individual paddocks ranging from 25 days in spring to 50 days in winter^22^. However, our data show that WF and WFG DM availability in P2 was significantly lower than in P1, indicating that regrowth is much lower than in PRG grazing systems. This is the first experiment conducted with growing beef steers in a rotational grazing system using willow fodder. In two similar experiments conducted in New Zealand with sheep, after topping the willow to stump level, 130 days and 60 days, respectively, were allowed before rotational grazing of WF occurred^23,24^. The study by Musonda et al.^25^ had a similar experimental design to this study. Similarly, during P2, each group grazed the same paddock area as in P1. The study used hoggets, and the length of time between paddock one in P1 and P2 was 83 days. While these studies did not measure WF herbage mass available or predicted FDMI between P1 and P2, it was noted that in the study by Musonda et al.^25^, the sheep had the same allocated paddock area between P1 and P2. However, the duration spent in each paddock varied between 5 and 14 days, depending on the WF available. This was similar to the present study, where steers had to be moved through weekly allocated WFG paddocks much faster (< 1 week) in P2 to prevent impacting FDMI and TDMI.

### Grazing willow silvopastoral systems reduce enteric CH_4_ emissions

Beef steers grazing the WFG treatment had a significant reduction of 27% in CH₄ relative to the PRG treatment, demonstrating tremendous potential to help meet cattle industry targets of a 30% reduction in CH₄ emissions by 2030^26^. The significant reduction in CH₄ observed in this study may be attributed to several key factors, such as DMI and levels of NDFI, both of which are linearly related to enteric CH₄ production^27–29^. In our study, the daily grazed forage DM intake (kg/d) was estimated from the difference between total ME requirement (MJ/d) and ME supply (MJ/d) from GF concentrate intake, divided by forage ME concentration. Moreover, it has been reported in the literature that animal diets with CT levels above 2–3% on a DM basis can be rejected by grazing ruminants due to the astringency of CTs, which makes them unpalatable^30^. In the present study, an overall CT inclusion of 5.65% showed that FDMI was not affected compared to the CT-free PRG treatment. Previous studies, such as Landau et al.^31^, observed no changes in feed intake when heifers were fed Quebracho and consumed 5% CTs on a DM basis. However, when CT inclusion amounted to 6.5% of DM intake, feed intake was impaired. Therefore, our findings indicated that the CT inclusion in our study was within the permissible limit to prevent FDMI reductions. However, our results also suggest that there is a specific threshold for including CTs in the diet before negative effects on feed intake occur. This threshold can vary depending on the plant source (Willow vs. Quebracho) and the structure of the CTs. Jones et al.^32^ found that the palatability of CTs containing Trifolium arvense and Trifolium affine was less than that of Coronilla varia, based on the order of their PD content. This is because CTs with a higher PD proportion have more H₂ bonding sites, potentially enhancing their affinity and bonding ability to fibre and protein^17^. In this study, the CTs had a higher proportion of PD relative to PC (PD: PC (%) = 71.1: 28.9), with no effect of the WFG forage on FDMI and TDMI. While this result showed no difference in FDMI with CTs of high PD content, our results are consistent with those found in the study by Lascano and Stewart^33^. Differing PC and PD contents of *Calliandra calothyrsus* CTs resulted in higher intakes and digestibility when PD was more dominant than PC in the extractable tannin fraction.

Regarding fibre intake, the WFG treatment had a significant 28% lower NDFI and a 21% greater ADFI. As a result, forages with higher NDF levels tend to produce more CH₄ during digestion^34^. Therefore, due to the lower NDFI intake of WFG, a reduced CH₄ production would be expected. In the present study, positive relationships between NDFI and ADFI with CH₄ production were observed, along with lower levels of CH₄ for the same NDFI and ADFI intake. Nevertheless, there was no distinction between WFG and PRG treatments for CH₄ production at the same NDFI. Therefore, it is suggested that the effect of CTs on reducing CH₄ emissions is greater than the effect of reductions in fibre digestibility.

The positive effects of CTs on reducing CH₄ production have been widely reported. In a similar study with willow, sheep were shown to have a 20% lower CH₄ emission per kg of metabolic body weight compared to control pasture with a CT content of 12 g/kg DM^24^. Furthermore, studies involving CTs containing *Lotus corniculatus (*LC), with a CT content of 8% DM, produced a 34% reduction in CH₄ yield compared to pasture^35^. In contrast to our observations, Beauchemin et al.^36^ found that by feeding beef steers a diet supplemented with quebracho tannin extract at a 1.8% inclusion level, no reductions in CH₄ emissions were observed. The authors concluded that CTs extracted from different plants vary widely in their capacity to bind carbohydrates and proteins. Additionally, this result might be due to the lower fibre content of the forages used rather than the presence of CTs. A similar example is the study by Carulla et al.^13^, who reported a 12% CH₄ reduction in sheep supplemented with the tannin extract Acacia mearnsii at a 2.5% CT inclusion. However, this CH₄ reduction was partially due to a 5% reduction in total tract NDF digestion. Moreover, Woodward et al.^37^ found that a group of dairy cattle grazing a CT-containing *sulla* forage produced less CH₄ (6.1% vs. 7.2% of GE intake) compared to a group grazing a perennial ryegrass (PRG) sward, where the NDF content of *sulla* was 70% lower than that of PRG (14.7% vs. 48.3% DM).

Two modes of action of CTs on methanogenesis have been proposed: (a) indirectly reducing fibre digestion, resulting in decreased H₂ production; and (b) directly inhibiting ciliate protozoa, fibre-degrading bacteria, and methanogenic archaea^14,18,38^. In the current study, H₂ production was non-significant between treatments, despite WFG showing a 22% numerical reduction. Therefore, it can be inferred that reductions in CH₄ could be attributed to the action of CTs on the rumen microbiome, inhibiting methanogenesis and microbial degradation. In vitro studies have shown that increasing CT inclusion decreases archaeal and protozoal populations, resulting in CH₄ reduction^39,40^. Furthermore, the structure of WFG CTs (higher in PD content and an mDP of 10.6) is conducive to reductions in CH₄ emissions, as seen in the literature, with Naumann et al.^21^ reporting that inhibitions of CH₄ correlate with increases in mDP and PD proportion. While this study did not analyse the impact of CTs on the rumen microbiome, it is suggested that the CTs in the present study may have manipulated the rumen microbiome, reducing its ability to carry out the redox chemistry required for CH₄ production.

Overall daily live weight gains are reduced by grazing willow silvopastoral systems. Overall, DLWG for WFG was significantly reduced by 31% relative to PRG. However, the reduction in DLWG on the WFG diet was primarily due to a much greater DLWG of steers grazing PRG in period 2 (PRG2). Despite no differences in nutritive intakes of N and ME between all treatments, it was expected that CT inclusion in the WFG treatment would result in higher DLWG relative to PRG. This is because CTs can bind to protein in the rumen, forming a complex that prevents degradation. As the complex travels to the abomasum, the pH drops, causing dissociation of the CT-protein complex and leading to greater delivery of true protein to the hindgut. This results in elevated amino acid uptake and increased weight gain^41^. In the study by Barry and McNabb^42^, a diet of LC with a CTI of 2.2% increased abomasal flow and net absorption of EAA as a proportion of N intake by 53% and 59%, respectively, with no effect on apparent digestibility.

Meanwhile, a diet of *Lotus penduculatus* with CTI 5.5% only increased abomasal flow and net absorption by 30% and 10% respectively. In their study, structural differences in CTs between these two treatments were insufficient and thus, it was suggested that the effects of the higher CTI in the *Lotus penduculatus* diet were due to the CTs not completely the protein into the small intestine. Moreover, in diets with a high CTI, once CTs are bound to proteins in the host plant and these proteins have been fully saturated, excess CTs can be released as a ‘free tannin,’ which can react with and inactivate microbial enzymes, reducing carbohydrate digestion^42^. This may explain the results in the current study, where DLWG did not increase with the WFG. The concentration of condensed tannins at 5.65% might be too high, preventing the effective release of protein complexes in the abomasum. Furthermore, the structure of the CT may come into play as different combinations of OH and H groups in these monomeric units lead to different classes of polymers, such as prodelphinidins (PD) and procyanidins (PC). Polymer composition affects CTs properties^43^ and affects how CTs influence feed digestion and nutritional value^44^. It is known that a low PD content decreases the ability of CTs to complex with proteins^45,46^. Therefore, it has been shown that a higher PC content (low PD content) of CTs in LC was associated with beneficial impacts on animal performance and amino acid absorption, compared to the higher PD content of CTs from *Lotus major* (Greater Birdsfoot Trefoil), which showed more astringent and anti-nutritional effects due to the higher PD content^47^.

In the present study, WFG CTs were more dominant in PD meaning they have a greater affinity for binding to protein and carbohydrates due to a greater number of H bonding sites with the potential to cause reductions in fibre degradation and acetate production in the rumen leading to reduced DLWG implications^48^. Molan et al.^49^ observed a higher PD content of CTs inhibited the growth of proteolytic bacteria. Thus, overall digestibility may have been negatively impacted for the cattle in this study meaning the benefits of CTs didn’t improve animal performance and DLWG.

An interesting observation from our research study was that the DLWG of beef steers under the PRG treatment in period 2 was significantly greater than that of the group of animals under the WFG treatment. This suggests that the CTI in WFG may have affected the ruminal microbiome. As the steers transitioned from WFG in P1 to PRG in P2, their ruminal microbiome may have been suppressed and less diverse^50^, leading to reduced rumen nutrient degradation by microbes. Consequently, more protein and energy may have reached the abomasum and small intestine, increasing the availability of amino acids for absorption. This could have resulted in compensatory growth for cattle in PRG2 transitioning from WFG1. Additionally, the improved nutrient supply in the lower digestive tract may have enhanced metabolic efficiency, further promoting tissue development and weight gain. Recent research has shown that low microbial richness in the rumen is closely linked to increased feed efficiency in dairy cows^51^. This study proposes that a reduction in rumen microbial richness in more efficient cows, results in a simpler metabolic network. This simplified network leads to higher concentrations of specific metabolites that support the host’s energy requirements, ultimately enhancing the cow’s ability to achieve greater daily live weight gain (DLWG). This suggests a more efficient conversion of feed into weight, where a less complex microbial community may contribute to more efficient energy utilization, promoting improved growth potential. In our study, the WFG treatment resulted in a significant 28% reduction in NDFI and a 21% increase in ADFI, which likely contributed to the observed 31% decrease in overall daily live weight gain in the WFG treatment compared to the PRG. Reduced NDFI limits the supply of digestible structural carbohydrates that promote rumination and efficient microbial fermentation, thereby reducing the production of volatile fatty acids that provide essential energy. At the same time, the increase in ADFI observed for the WFG treatment, results in a higher proportion of indigestible fibre (mainly cellulose and lignin), and impair live weight gain and overall animal performance.

### Faecal samples of steers grazing willow silvopastoral systems do not contain intact condensed tannins 

The two-dimensonal NMR spectra of faecal samples from WFG1 and WFG2 show no presence of intact CT. This suggests that the colour produced from the bound fraction of the sequential HBAI assay is not from intact CT but may be derived from CT metabolites arising from CT compositionally altered during digestion which retain the capability of being oxidized to a coloured pigment. The overall outcome of CT lost during ruminant digestion and subsequent metabolic pathways remain to be determined^52^. The study of Terrill et al. (1994)^53^ used ^14^C-labelled CT to measure the digestion of CT contained in *Lotus pedunculatus* (20–100 g CT/kg DM) along the rumen, abomasa, ilea digesta and faecal samples which varies in PC/PD ratio from 16/84 to 29/71^20,54^. The CT content detected was low across all digesta samples, demonstrating the disappearance of CT across the rumen, abomasum and small intestine, while CT recovery in faeces was reported to be 15% of CT intake. Many other studies have shown that CT can remain intact in the faeces after digestion. For example, a study involving feeding CT containing *Sericea Lespedeza* with alfalfa forage at a CTI of 7.28% resulted in the faeces containing 3.8% of the total CT fed^52^ showing nearly all CT (bound and unbound) were compositionally altered to non-proanthocyanidin structures. The CT structure of *Sericea Lespedeza* had a (PD/PC) ratio of 94/6, a cis/trans ratio of 79/21, and a mDP of 13.7. Alternatively, quebracho CT, with a different structure also was found to survive digestion by sheep^55^.

With one notable exception^52^, it is important to point out that previous studies reporting the presence of CTs in ruminant faeces rely solely on using the HCl-butanol assay to assess CT content. In the current study, the λ_max_ of the non-CT components giving rise to the purple colour, is 551 nm, very close to the reported λ_max_ of cyanidin and delphinidin generated from procyanidin and prodelphinidin (555 and 560 nm, respectively) flavan-3-ol subunits present in the intact CT. Thus, it is quite possible that the slight hypsochromic shift may have been understandably overlooked during CT assessment via the HCl-butanol assay. This observation serves as a cautionary note that researchers should be aware that results from the HCl-butanol assay may be tainted and that non-CT or metabolized CT components, and not intact CT, may be giving rise to the colouration in faecal samples similar to that seen in the generation of cyanidin and delphinidin ions.

While none of the previous studies have measured CH_4_ production from the rumen compared with the present study, the findings of Becker et al.^49^ provide an explanation as to why no intact CTs were found in the faeces of the steers. In vitro rumen fermentation studies have shown that the CT flavan-3-ol subunit catechin can be metabolized into a series of phenolic acids, effectively serving as a hydrogen sink, contributing to the inhibition of ruminal CH_4_ production^56^.The mixture of CTs present in the rumen, already a collection of literally millions of unique CT chemical entities, could be reduced at any individual flavan-3-ol subunit along the chain of the innumerable oligomers and polymers, would generate an exponential number of reactive reduction product. The multitude of products from degradations of this type would be extremely challenging to detect and quantify. These reactive products may also crosslink with other components of the digesta or may be further altered metabolically and excreted in the urine.

## Materials and methods

All animal measurements were carried out under the regulations of the Department of Health, Social Services and Public Safety of Northern Ireland in accordance with the Animals (Scientific Procedures) Act 1986. All procedures adopted in the present experiments were approved by the Ethical Review Committee of the Agri-Food and Biosciences Institute (Hillsborough, United Kingdom) and were in accordance with the UK Animal Scientific Procedures Act (1986). The experimental protocols were conducted in accordance with the relevant guidelines and regulations. The study design and analysis conform to the ARRIVE recommendations for animal research (https://arriveguidelines.org).

### Experimental design and grazing management

Twenty, 2021 born Aberdeen Angus sired X Holstein Friesian Steers were used in a two-treatment (grazing swards) by two period Latin square design study. The cattle had a mean initial body weight (BW) of 494 kg (s.d. 42.9 kg) and were balanced across the two treatments according to BW, and age. All cattle received anthelmintic treatment before the trial commenced using an ivermectin pour-on (Noromectin pour-on, Norbrook, Newry, UK).

The two swards were a perennial ryegrass-grazing platform (PRG) at Foyle Farms of Excellence, Cookstown, UK, and a willow fodder mix with a grass understory (WFG) at Brook Hall Estate, Londonderry, UK. The PRG site covered 2 ha of predominantly perennial ryegrass (*L. perenne L*.). The WFG site covered 4 ha and comprised a polyclonal mix of willow species consisting of *Salix Ashton Stott*,* Salix Parafitt*,* Salix Tora*,* Salix Sven*,* Salix Torhilde and Salix Olaf* alongside a grass understory mainly made up of perennial ryegrass (*L. perenne L*.). The PRG site was subdivided into four different blocks (0.5 ha each) containing a GreenFeed (GF) machine and Ritchie Water weigh scale (WWS) placed in the centre. Cattle at the PRG site were rotated ~ every 2 weeks. Similarly, at the WFG site, cattle were allocated a weekly block (~ 0.38 ha each) with the GF and WWS located at the end on each block. Seven weekly blocks were allocated at WFG site. Different site and weekly block sizes were due to the different growth rates of PRG and WFG.

Grazing at each site commenced on 7 June 2022 and continued until 26 August 2022 (80 days), including two experimental periods At the end of each period, each group of cattle was re-allocated to the other treatment and transported between sites on the same day. No supplementary feeding was offered, except for a maximum of 1.4 kg/d (fresh weight) of concentrate pellets offered as bait for the GF measurements. All cattle accessed water *ad libitum* through the WWS allowing a daily average weight to be recorded. Cattle on the PRG treatment were rotationally grazed around each block biweekly while cattle on the WFG treatment were allocated a weekly block and moved to the consecutive measured out block. Willow fodder regrowth was assessed, and it was observed that after 7 weeks (7 blocks) cattle were able to re-graze the original weekly grazing blocks grazed at the start of the trial. During this re-graze phase (P2) cattle on the WFG in some instances had to be moved through some blocks in less than 7 days due to limited fodder. The first fourteen days of each period were considered the adaptation period.

### Willow fodder and grass understory forage dry matter yield 

To determine the total forage DM available for cattle on the WFG treatment, four weekly the DM (kg) of WF and GU were measured at three random 1 m^2^ plots allowing a DM yield/m^2^ to be calculated. The total area of each WFG block was determined using GPS: Fields Area Measure (Rento UAB, Lithuania) allowing calculation of the total DM yield of forage available on each weekly block of WFG. This method was adapted from Kemp et al. (2019)^57^.

### Feed sampling and analysis

Weekly samples of perennial ryegrass from the PRG treatment and Willow fodder (WF) and Grass understory (GU) from the WFG treatment were collected. The collected material was subjected to a drying process at a temperature of 60^0^C for 48 h followed by grinding to a particle size of 1 mm and kept at room temperature. The samples were analysed for dry matter (DM), ether extract (EE), and ash according to AOAC official methods^58^. Acid detergent fibre (ADF) and neutral detergent fibre (NDF), according to Goering and Van Soest (1970)^59^, were performed by the ANKOM 220 Fiber Analyser (ANKOM Technology Corporation, New York, USA) with sodium sulphite and heat stable α-amylase. Nitrogen content in the samples was analysed via the Dumas method^60^, with the Leco Protein Vario Max CN analyser (Elementar Analysensysteme, GmbH, Hanau, Germany) and crude protein (CP) calculated using N x 6.25. Gross energy (GE) content was determined by AFBI Hillsborough Analytical Services using an isothermal automated bomb calorimeter (PARR, Instrument Model 6300, UK). An additional weekly fresh sample of PRG was analysed using near-infrared spectroscopy (NIRS) for DM, CP, ADF, NDF, water-soluble sugars (WSC; %DM), and metabolisable energy (ME; MJ/kg DM).

Concentrate samples were collected three times during each period from both GF systems. Samples were analysed for DM, GE, N, CP, Ash, EE, NDF and ADF determinations. Starch concentrations were determined using a total starch assay kit (Megazyme International Ireland Ltd, Wicklow, Ireland). The laboratory analyses were performed in triplicate.

### Purification of the willow fodder CTs and willow faecal reference standard 

The willow fodder CTs reference standard for period 1 was obtained following the procedures of Brown et al. and Naumann et al.^21,61^. Ground willow fodder leaf material (50 g) was placed in a 1000 mL Erlenmeyer flask equipped with a magnetic stir bar and diluted with acetone/water (7:3, 500 mL). The mixture was rapidly stirred for 30 min and then filtered through a glass-sintered funnel equipped with filter paper (Reeve Angel, grade 202). The residue was returned to the Erlenmeyer flask stirred with fresh acetone/water (7:3, 500 mL), and filtered two additional times. The combined three acetone/water extracts were concentrated under reduced pressure (rotary evaporation) at ≤ 40 °C to remove the acetone and the resulting aqueous layer was stirred with ethyl acetate (400 mL) overnight. The aqueous layer was separated using a separatory funnel and stirred a second time with ethyl acetate (400 mL) for about 2 h. The aqueous layer was separated and placed under reduced pressure (rotary evaporation) at ≤ 40 °C to remove any traces of ethyl acetate and then freeze-dried to give 8.754 g of extract. This extract was diluted with methanol/water (1:1, 100 mL) and Sephadex LH-20 (GE Healthcare, Uppsala, Sweden) in small portions while stirring with a spatula, until the mixture reached the consistency of wet sand (32.6 g of Sephadex LH-20 added). The CT-laced resin was transferred to a 500 mL sintered glass funnel equipped with filter paper (Reeve Angel, grade 202). The resin was suspended in methanol/water (1:1, 200 mL), allowed to stand for ~ 5 min, and then vacuum filtered. This methanol/water washing of the resin was repeated 14 additional times. The resin was then suspended in acetone/water (7:3, 200 mL), allowed to stand for ~ 5 min, and then vacuum filtered. This acetone/water washing of the resin was repeated three additional times. The four acetone/water washings were combined and concentrated under reduced pressure (rotary evaporation) at ≤ 40 °C to remove the acetone and freeze-dried to give 1.36 g of an off-white solid, sufficiently pure to serve as the WF CT reference standard in this study.

The WF CT reference standard for period 2 was obtained in the exact same manner with the following changes: the amount of initial extract obtained, 8.39 g; Sephadex LH-20 mass used, 31.557 g; mass of freeze-dried purified willow fodder CTs reference standard from period 2, 1.31 g.

### NMR spectroscopy

^1^H, ^13^C, and ^1^−^13^ C HSQC NMR spectra for the purified willow fodder CTs (period 1 and 2) were recorded at 27 °C on a BrukerBiospin DMX-500 (^1^H 500.13 MHz, ^13^C 125.76 MHz) instrument equipped with TopSpin 3.5 software and a cryogenically cooled 5 mm TXI ^1^H/^13^C/^15^N gradient probe in inverse geometry. Spectra were recorded in DMSO-*d*_*6*_ and were referenced to the residual signals of DMSO-*d*_*6*_ (2.49 ppm for ^1^H and 39.5 ppm for ^13^C spectra). For ^1^−^13^ C HSQC experiments, spectra were obtained using the standard Bruker pulse program HSQC gtpsi.

### Preparation of HCl-butanol-acetone-iron (HBAI) assay solution for sequential CTs content

The reaction medium was prepared fresh daily on a per 200 mL basis by first dissolving 333 mg of ammonium iron (III) sulfate dodecahydrate in 11 mL of concentrated HCl (37%, w/v) and then adding 93 mL of 1-butanol, followed by 96 mL of acetone with stirring; then 13.5 mL of this solution was mixed with 1.5 mL of acetone − water in all tubes, this yielded a final reaction medium formulation similar to that used in the direct assay of Grabber et al. (2013)^54^.

### Sequential determination of CTs content

CT content determinations were performed in triplicate runs on different days and determinations are averages of triplicate analyses and repeated three times. The sample, previously ground to pass through a 1 mm screen, was briefly ball-milled for 2 min to provide a more homogenous sample. Approximately 20 mg (to the nearest tenth of a mg) of WF samples were weighed into Teflon-capped 30 mL capacity thick-walled, screw-capped glass centrifuge tubes (e.g., Kimble 45600-30). A mixture of acetone-water (7:3, 15 mL) was added to each of the tubes, the tubes were capped and subjected to sonication (Bronsen 8510 sonicator) for 45 min with gentle mixing every 15 min to resuspend the tissue. The tubes were then centrifuged at 6000 x g for 20 min at room temperature, and subsamples of acetone-water extract supernatants (1.5 mL) were transferred to Teflon-capped 25 mL capacity thick-walled glass tubes. The remaining portion of the supernatants were carefully removed via a pipette without disturbing insoluble residue pellets. Next, 1.5 mL of 7:3 acetone-water was added to insoluble residues in centrifuge tubes and to CTs standards (0.0, 0.025, 0.050, 0.075, 0.100, and 0.125 mL of a 5.00 mg/mL solution of the CTs reference standard corresponding to 0.00, 0.125, 0.250, 0.375, 0.500, and 0.625 mg, respectively) in Teflon capped 25 mL capacity thick-walled glass tubes. The HBAI assay solution (13.5 mL) was added to all tubes. The screw caps were added to the test tubes and the tubes were heated in an aluminium block at 70 °C for 3 h. Every 15 min the tubes were removed and briefly vortexed to ensure proper mixing during the reaction. After cooling to room temperature over about 1 h, 2 mL aliquots were removed from each of the triplicate runs, placed in 2 mL conical polypropylene-copolymer microcentrifuge tubes, sealed with screw caps, and centrifuged for 5 min at 10 000 g. After centrifugation, the clarified supernatants were scanned with a Shimadzu UV-2600 spectrophotometer (Shimadzu Scientific Instruments, Columbia, MD) using the Shimadzu UVProbe version 2.43 software package from 400 to 600 nm, and the maximal absorption at λ_max_ of the anthocyanidin peak was recorded. The absorbance data was corrected for the small dilution factor of the reference standards and for the purity of the reference standard. The same method applied for the faecal samples, with the exception individual faecal samples of all cattle grazing WFG were pooled into one sample. Representative faecal samples for P1 and P2 were performed in triplicate runs and repeated two times.

### GreenFeed measurements

GreenFeed (GF system, C-Lock, Inc., South Dakota, USA), a portable open circuit head system was used to measure O_2_ consumption and CO_2_, CH_4,_ and H_2_ production during the full experimental period. The system includes animal identification, feed dispensing, air handling, gas measurement, gas calibration, electronics, communication, and data handling systems. In the GF system a fan draws air past the animal’s muzzle into a duct system where airflow rates are monitored, and samples are taken to determine the concentrations of CH_4_ and CO_2_. The GF system includes several calibration mechanisms to ensure proper airflow, gas sensor functionality, air recovery, and the animal’s head position. Emissions are calculated based on the airflow and gas concentrations detected by the sensors, with adjustments made for background levels. Since most methane is released during eructation that happen every 1–2 min, the animal must keep its head in the hood for at least 2 min to obtain an accurate measurement. In our study a pelletized concentrate bait (Growing cattle, Fane Valley Feeds, UK) was offered to entice the cattle to visit the GF system. The system was configured to allow cattle to visit at a minimum of 5-h intervals. During each visit, cattle were given 8 separate deliveries of bait concentrate pellets (40 g per delivery), once every 40 s. The average concentrate bait intake from the GF system was 0.788 kg DM/d. The system also recorded muzzle position during the visits and data with inappropriate muzzle positions were removed.

One animal in PRG1 did not attend the GF regularly (> 5 h intervals) enough to obtain gaseous exchange measurements set by GF system (C-Lock) and was removed from all other subsequent calculations and statistics.

### Live weight gain measurements 

Steers were weighed by the WWS (Ritchie, Angus, Scotland, UK) with one placed at each experimental treatment site. Cattle accessed water through this system acting as bait to weigh the cattle. The WWS system was calibrated at the start of the trial. A daily average weight was produced for each day cattle accessed the WWS system. A mean BW was calculated for each period and the daily live weight gain (DLWG) of each animal over the two different treatments was calculated from the slope of the linear regression of BW against time. Any outlier weights were removed before the calculation of linear regression.

### Heat Production and prediction grazed forage intake

Heat production (HP) was calculated according to Brouwer^62^ using volumes of O_2_ consumption (L/d), CH_4_ and CO_2_ production (L/d) from the GF system and estimated urinary nitrogen (UN) excretion from total N intake according to Angelidis et al. (2021)^63^. Methane energy (CH_4_-E; MJ/d) was calculated by the factor 0.05524 MJ/g of CH_4_ proposed by Kriss^64^. Daily grazed forage DM intake (kg/d) was estimated from the difference between total ME requirement (MJ/d) and ME supply (MJ/d) from GF concentrate intake divided by forage ME concentration (MJ/kg DM). Total ME requirement (MJ/d) was a sum of HP (MJ/d) and net energy required to produce daily live weight gain which was calculated according to AFRC^65^. Grass ME concentration was predicted according to NIRs as described previously, while ME concentrations of WF and GU were estimated from the procedure of Menke and Steingass using gas production data^66^. In addition, concentrate ME concentration was calculated according to Boguhn et al.^67^.

### Faecal condensed tannin content and structure

Forty faecal samples (10 steers x 2 periods x 2 treatments) were spot sampled and stored at -80 °C. After thawing, samples were dried at 30 °C for 72 h and ground to 1 mm. The 40 faecal samples were then pooled according to treatment and period giving representative samples: PRG1, WFG1, PRG2 and WFG2. These four samples underwent similar analysis for CTs content and structure as described for the willow fodder samples.

### Statistical analysis

Data were analysed using two-way Anova and Kruskal-Wallis for evaluation of the effects of forage treatment and period on animal and forage measurements. Post hoc comparisons were carried out using Pairwise t-test to calculate the least significant differences among the means. The normality of residuals was performed using the Shapiro-Wilk test, and homogeneity of variance using the Levene Test allowing the correct statistical test to be applied to each residual. All data were analysed R Studio^68^. Statistical differences were considered significant at *P* < 0.05 and as a tendency at 0.05 ≤ *P* < 0.10.

## Conclusion

Introducing willow fodder into the diet of steers offers a viable solution for enhancing sustainable beef production, achieving a 27% reduction in CH4 emissions (g/d) for WFG relative to PRG. This study highlights the significant potential of combining agroforestry practices with rotational grazing systems to not only reduce above-ground carbon emissions but also improve below-ground carbon sequestration. By demonstrating willow’s ability to decrease GHG emissions within ruminant systems, this research contributes to the development of climate-smart agricultural practices. Practically, these findings suggest that integrating willow fodder into beef production systems can help meet emissions reduction targets, improve overall sustainability, paving the way for more environmentally responsible and resource-efficient production methods.

## Electronic supplementary material

Below is the link to the electronic supplementary material.

Supplementary Material 1

## Data Availability

All data supporting this study is provided as supplementary information accompanying this paper.
