# Supplementary material for: Willow silvopastoral systems as a strategy to reduce methane emissions while maintaining cattle performance
Source: Sci Rep. 2025 Jun 2;15:19310. doi: 10.1038/s41598-025-02289-0 (PMC12130242; doi:10.1038/s41598-025-02289-0)
Supplement: Supplementary file 1 — Supplementary Material 1 [file 41598_2025_2289_MOESM1_ESM.docx]

**Supplementary Material for:**

**Willow Silvopastoral Systems as a Strategy to Reduce Methane Emissions While Maintaining Cattle Performance**

Joshua Philip Thompson, Stergiadis Stergiadis, Omar Cristobal Carballo, Wayne E. Zeller, Tianhai Yan, Francis Lively, John Gilliland, Rudra N Purusottam, Sharon Huws, Katerina Theodoridou

**Table of Contents**

**Table S 1.** Nutritional composition of all forages and concentrates used in this study and structural analysis of condensed tannins…………………………………………..45

**Table S 2.** Animal performance, forage and nutritional intakes of beef steers ……..46

**Table S 3.** Gaseous exchange of steers, heat production estimates, and methane ratios…………………………………………………………………………………………47

**Figure S 1.** ^1^H-^13^C HSQC NMR spectrum of the Willow fodder reference standard from period 1 showing the cross-peak signals used to assess the purity of the sample………………………………………………………………………………………48

**Figure S 2.** ^1^H-^13^C HSQC NMR spectrum of the Willow fodder reference standard from Period 2 showing the cross-peak signals used to assess the purity of the sample………………………………………………………………………………………48

**Figure S 3.** ^1^H-^13^C HSQC NMR spectrum of pooled faecal sample of steers grazing Willow fodder in period 1……………………………………………………………….…49

**Figure S 4.** ^1^H-^13^C HSQC NMR spectrum of pooled faecal sample of steers grazing Willow fodder in period 2……………………………………………………………….…49

**Figure S 5.** An expanded section of Figure S 4 ^1^H-^13^C HSQC NMR spectrum emphasizing the lack of both the C-6/8 and C-2',6' C-H cross-peaks signals in faecal samples…………………………………………………………………………………..…50

**Figure S 6.** Wavelength of pooled faecal sample of steers grazing WFG in period 1 compared to the WF CT reference standard………………………………………..…51

**Figure S 7.** Wavelength of pooled faecal sample of steers grazing WFG in period 1 compared to the WF CT reference standard………………………………………..…51

Table S 1. Nutritional composition of all forages and concentrates used in this study and structural analysis of condensed tannins.

|  | Nutritional Composition | | | |
| --- | --- | --- | --- | --- |
|  | PRG | WF | GU | C |
| DM (g/kg fresh) | 232 | 289 | 214 | 903 |
| Ash (g/kg DM) | 77.8 | 72.7 | 80.6 | 95.2 |
| ADF (g/kg DM) | 285 | 305 | 319 |  |
| NDF (g/kg DM) | 566 | 325 | 516 |  |
| Starch (g/kg DM) |  |  |  | 165 |
| EE (g/kg DM) | 28.5 | 25.3 | 27.6 | 40.4 |
| N (g/kg DM) | 28.7 | 28.4 | 17.8 | 31.1 |
| CP (g/kg DM) | 179 | 178 | 111 | 195 |
| GE (g/kg DM) | 18.6 | 19.9 | 18.6 | 18.1 |
| ME (g/kg DM) | 10.7 | 8.95 | 9.4 | 12.6 |
|  |  |  |  |  |
| Condensed tannins |  |  |  |  |
| CT (g/kg DM) | - | 77.8 | - | - |
| mdp | - | 10.6 | - | - |
| PC (%) | - | 28.9 | - | - |
| PD (%) | - | 71.2 | - | - |
| Cis (%) | - | 23.4 | - | - |
| Trans (%) | - | 76.7 | - | - |
|  |  |  |  |  |
| PRG, perennial rye grass; WF, willow fodder; GU, grass understory; C, concentrates; DM, dry matter; ADF, acid detergent fibre; NDF, neutral detergent fibre; EE, ether extract; N, nitrogen; CP, crude protein; GE, gross energy; ME, metabolisable energy; CT, condensed tannins; mDP, mean degree of Polymerisation; PC, procyanidin; PD,Prodelphinidin | | | | |

Table S 2. Animal performance, forage and nutritional intakes of beef steers.

|  |  | **Forage** | | | **Period** | | | **Interaction** | | | | | |
| --- | --- | --- | --- | --- | --- | --- | --- | --- | --- | --- | --- | --- | --- |
|  |  | **PRG** | **WFG** | **P-value** | **1** | **2** | **P-value** | **PRG1** | **WFG1** | **PRG2** | **WFG2** | **s.e.m.** | **P-value** |
| Row |  | (n=19) | (n=20) |  | (n=19) | (n=20) |  | (n=9) | (n=10) | (n=10) | (n=10) |  |  |
| 1 | **Animal Performance** | | |  |  |  |  |  |  |  |  |  |  |
| 2 | **LW (kg)** | 507 | 513 | 0.525 | 486 | 533 | *** | 476 | 495 | 535 | 531 | 6.01 | 0.225 |
| 3 | **LWG (kg/d)** | 1.04 | 0.720 | ** | 0.760 | 0.990 | * | 0.764^a^ | 0.748^a^ | 1.29^b^ | 0.685^a^ | 0.0605 | ** |
|  |  |  |  |  |  |  |  |  |  |  |  |  |  |
| 4 | **Feed DMI (kg/DM)** | | |  |  |  |  |  |  |  |  |  |  |
| 5 | **CDMI** | 0.700 | 0.750 | 0.757 | 0.440 | 0.990 | *** | 0.304 | 0.570 | 1.05 | 0.928 | 0.0548 | *** |
| 6 | **FDMI** | 9.46 | 10.2 | 0.100 | 10.4 | 9.29 | ** | 9.92 | 10.9 | 9.06 | 9.52 | 0.229 | 0.960 |
| 7 | **TDMI** | 10.2 | 11.0 | 0.0591 | 10.9 | 10.3 | 0.164 | 10.2 | 11.5 | 10.1 | 10.5 | 0.217 | 0.274 |
| 8 |  |  |  |  |  |  |  |  |  |  |  |  |  |
| 9 | **Total Nutrient intake** | | |  |  |  |  |  |  |  |  |  |  |
| 10 | **GEI (MJ/d)** | 189 | 213 | ** | 207 | 196 | 0.193 | 189 | 223 | 188 | 203 | 4.42 | 0.234 |
| 11 | **MEI (MJ/d)** | 110 | 102 | 0.0728 | 106 | 105 | 0.752 | 106 | 107 | 113 | 97.4 | 2.17 | 0.0607 |
| 12 | **ADFI (g/d)** | 2700 | 3280 | *** | 3160 | 2840 | * | 2940 | 3360 | 2480 | 3200 | 80.3 | 0.244 |
| 13 | **NDFI (g/d)** | 5360 | 3840 | *** | 4790 | 4380 | * | 5470 | 4170 | 5250 | 3500 | 162 | 0.283 |
| 14 | **SI (g/d)** | 102 | 126 | 0.177 | 78.0 | 150 | *** | 55.1^a^ | 98.6^b^ | 145^c^ | 154^c^ | 7.76 | *** |
| 15 | **NI (g/d)** | 294 | 285 | 0.844 | 252 | 324 | *** | 212^a^ | 289^b^ | 368^c^ | 280^b^ | 10.4 | *** |
| 16 | **CT (g/d)** | 0.00 | 617 | *** | 306 | 326 | 0.570 | 0^a^ | 582^b^ | 0^a^ | 652^c^ | 50.9 | *** |
| 17 | **CTI (%DM)** | 0.00 | 5.65 | *** | 2.66 | 3.12 | 0.179 | 0^a^ | 5.06^b^ | 0^a^ | 6.23^c^ | 0.463 | *** |
|  |  |  |  |  |  |  |  |  |  |  |  |  |  |
| PRG, perennial rye grass; WFG, willow fodder grass; LW, liveweight; LWG, liveweight gain; DMI, dry matter intake; CDMI, condensed tannin intake; FDMI, forage DM intake; TDMI, total dry matter intake; GEI, gross energy intake; MEI, Metabolisable energy intake; ADFI, acid detergent fibre intake; NDFI, neutral detergent fibre intake; SI, starch intake; NI, nitrogen intake; CTI, condensed tannin intake | | | | | | | | | | | | | |

Table S 3. Gaseous exchange of steers, heat production estimates, and methane ratios.

|  |  | **Forage** | | | **Period** | | | **Interaction** | | | | | |
| --- | --- | --- | --- | --- | --- | --- | --- | --- | --- | --- | --- | --- | --- |
|  |  | **PRG** | **WFG** | **P-value** | **1** | **2** | **P-value** | **PRG1** | **WFG1** | **PRG2** | **WFG2** | **s.e.m.** | **P-value** |
| Row |  | (n=19) | (n=20) |  | (n=19) | (n=20) |  | (n=9) | (n=10) | (n=10) | (n=10) |  |  |
| 1 | **Gaseous Exchange (g/d)** |  |  |  |  |  |  |  |  |  |  |  |  |
| 2 | **CO_2_** | 7790 | 7720 | 0.844 | 8230 | 7300 | *** | 8430^a^ | 8050^a^ | 7210^b^ | 7390^b^ | 128 | ** |
| 3 | **O_2_** | 5570 | 5750 | 0.250 | 5960 | 5380 | *** | 5880 | 6030 | 5280 | 5470 | 89.9 | 0.876 |
| 4 | **H_2_** | 1.38 | 1.08 | 0.0877 | 1.29 | 1.17 | 0.448 | 1.41 | 1.18 | 1.36 | 0.975 | 0.0878 | 0.676 |
| 5 | **CH_4_** | 237 | 173 | *** | 215 | 194 | * | 254 | 180 | 221 | 167 | 7.18 | 0.332 |
| 6 |  |  |  |  |  |  |  |  |  |  |  |  |  |
| 7 | **Heat Production (MJ/d)** | 82.3 | 84.3 | 0.384 | 88.0 | 78.8 | *** | 87.8 | 88.3 | 77.4 | 80.3 | 1.33 | 0.599 |
| 8 |  |  |  |  |  |  |  |  |  |  |  |  |  |
| 9 | **CH_4_/LW (g/kg)** | 0.470 | 0.340 | *** | 0.444 | 0.365 | *** | 0.535^a^ | 0.364^b^ | 0.413^bc^ | 0.316^b^ | 0.0152 | * |
| 10 | **CH_4_/LWG (g/kg)** | 269 | 296 | 0.555 | 322 | 245 | * | 374^a^ | 276^a^ | 174^b^ | 317^a^ | 21.3 | ** |
| 11 | **CH_4_-E (MJ/d)** | 13.1 | 9.58 | *** | 11.9 | 10.7 | * | 14.0 | 9.95 | 12.2 | 9.21 | 0.397 | 0.332 |
| 12 | **CH_4_-E/GEI (%)** | 6.95 | 4.51 | *** | 5.89 | 5.52 | * | 7.46^a^ | 4.47^b^ | 6.49^c^ | 4.54^bd^ | 0.227 | * |
|  |  |  |  |  |  |  |  |  |  |  |  |  |  |
| CO_2_, carbon dioxide; O_2_, oxygen; H_2_, hydrogen; CH_4_, methane; LW, liveweight; LWG, liveweight gain; E, energy; GEI, gross energy intake | | | | | | | | | | | | | |


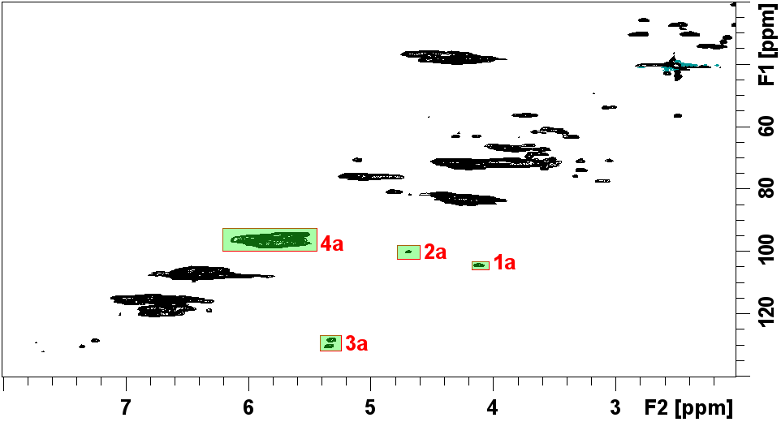


Figure S 1^1^H-^13^C HSQC NMR spectrum of the Willow fodder reference standard from period 1 showing the cross-peak signals used to assess the purity of the sample. Impurity cross-peaks from C-1 H/C beta (Area 1a) and alpha (Area 2a) anomeric signals and the H/C cross-peaks arising from the carbon-carbon olefin double bonds in the lipid impurities present (Area 3a) were compared to the C-6/8 H/C cross-peak signals (Area 4a) from the A ring of the CT present are highlighted.


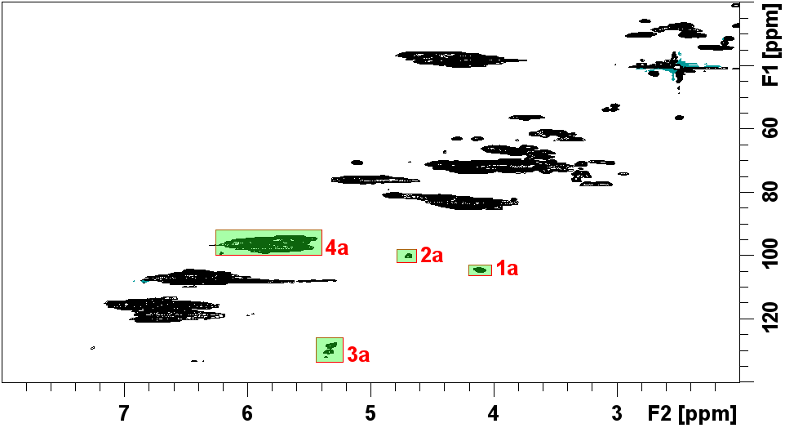


Figure S 2. ^1^H-^13^C HSQC NMR spectrum of the Willow fodder reference standard from Period 2 showing the cross-peak signals used to assess the purity of the sample. Impurity cross-peaks from C-1 H/C beta (Area 1a) and alpha (Area 2a) anomeric signals and the H/C cross-peaks arising from the carbon-carbon olefin double bonds in the lipid impurities present (Area 3a) were compared to the C-6/8 H/C cross-peak signals (Area 4a) from the A ring of the CT present are highlighted.


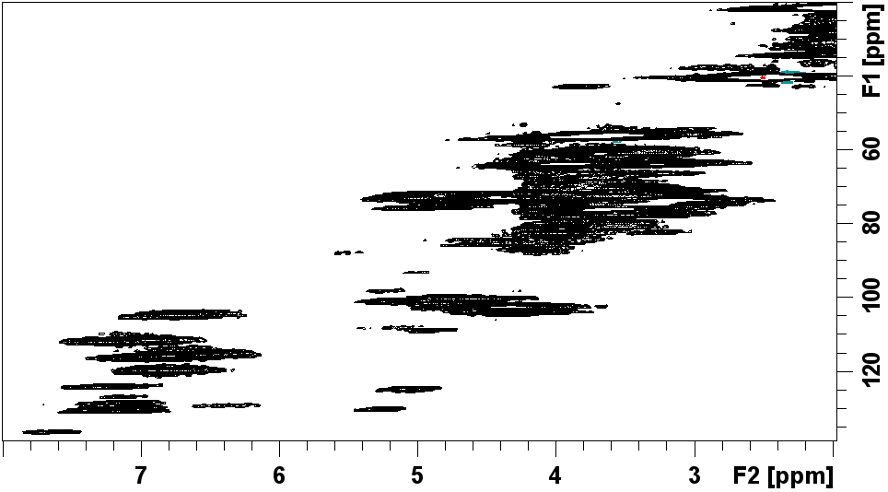


Figure S 3. ^1^H-^13^C HSQC NMR spectrum of pooled faecal sample of steers grazing Willow fodder in period 1.


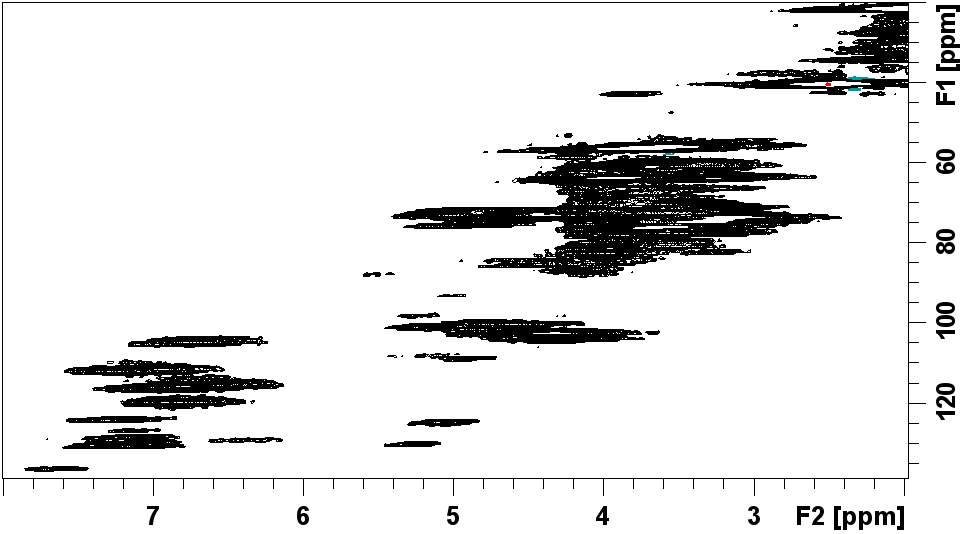


Figure S 4. ^1^H-^13^C HSQC NMR spectrum of pooled faecal sample of steers grazing WFG in period 2.


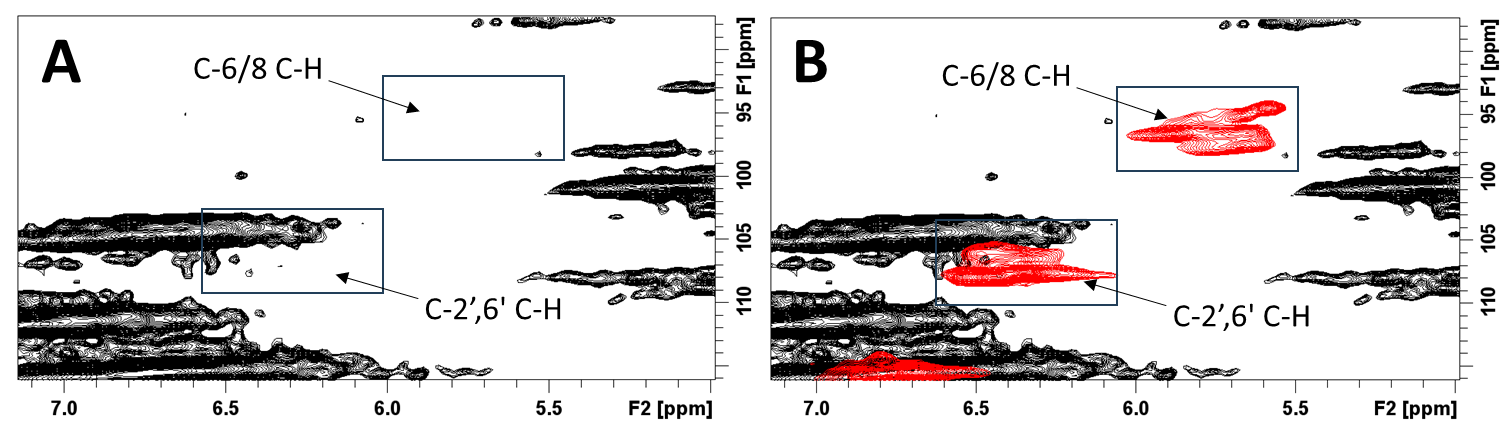


Figure S 5. An expanded section of Figure S 4 ^1^H-^13^C HSQC NMR spectrum emphasizing the lack of both the C-6/8 and C-2',6' C-H cross-peaks signals (Panel A) in faecal samples from P2. Panel B shows this section of the spectrum overlapped with the spectrum of the CT reference standard of willow fodder from period 2.


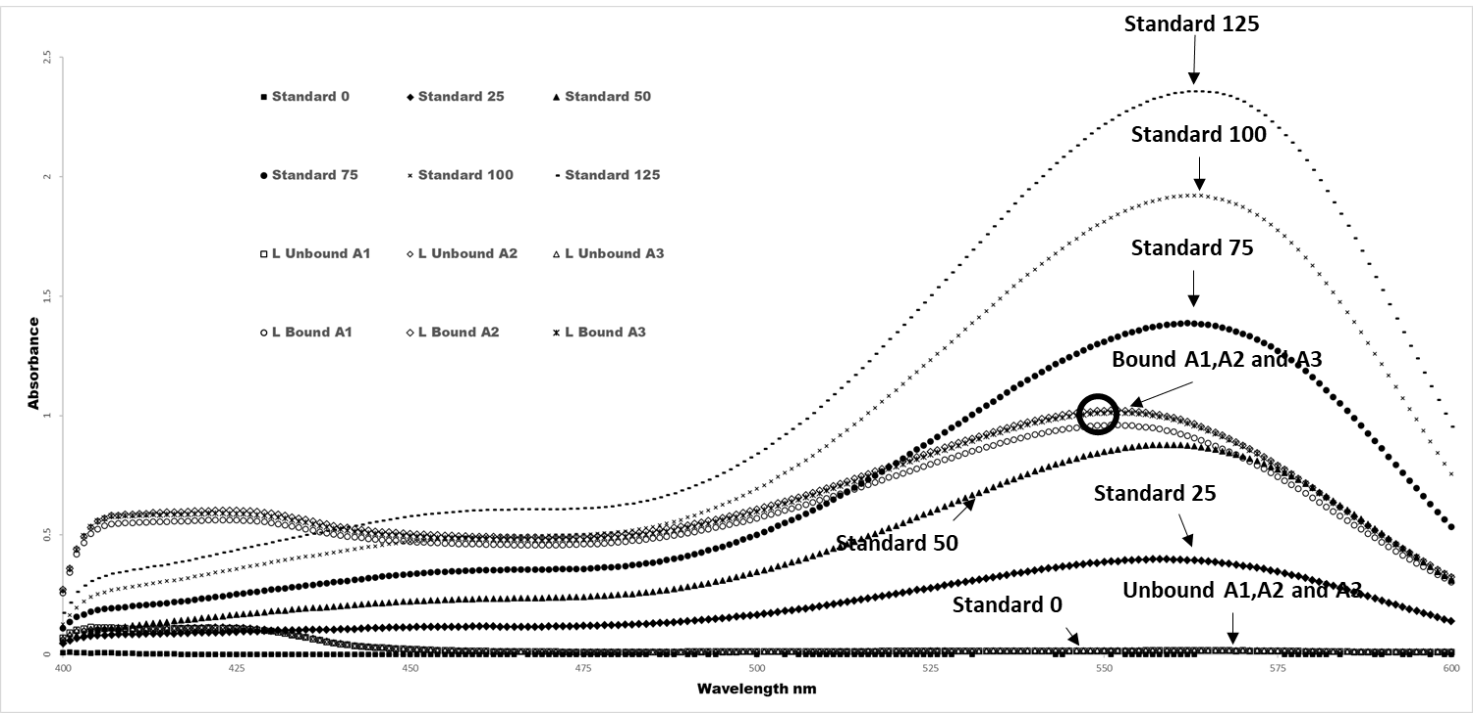


Figure S 6. Wavelength of pooled faecal sample of steers grazing WFG in period 1 compared to the WF CT reference standard.


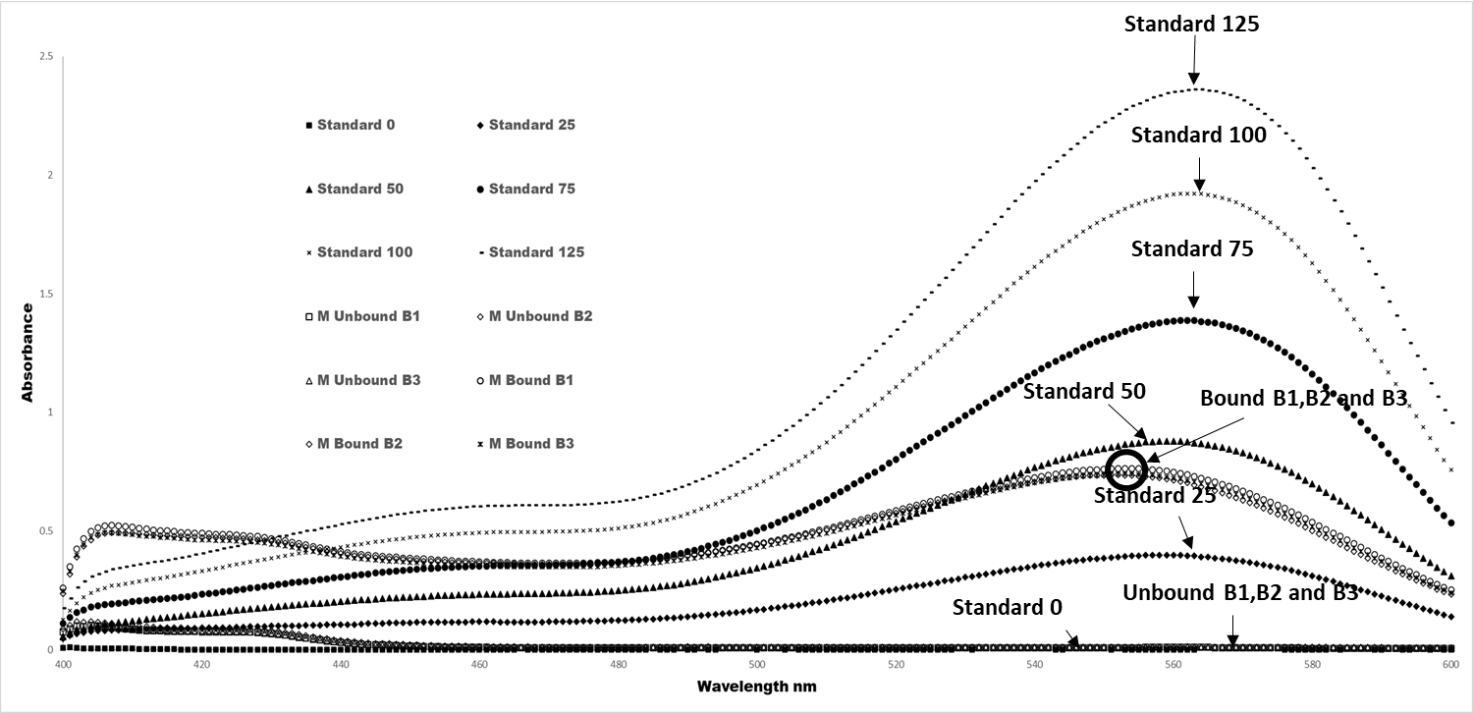


*Figure S 7. Wavelength of pooled faecal sample of steers grazing WFG in period 2 compared to the WF CT reference standard.*
